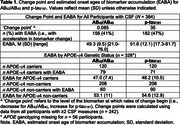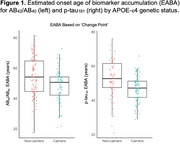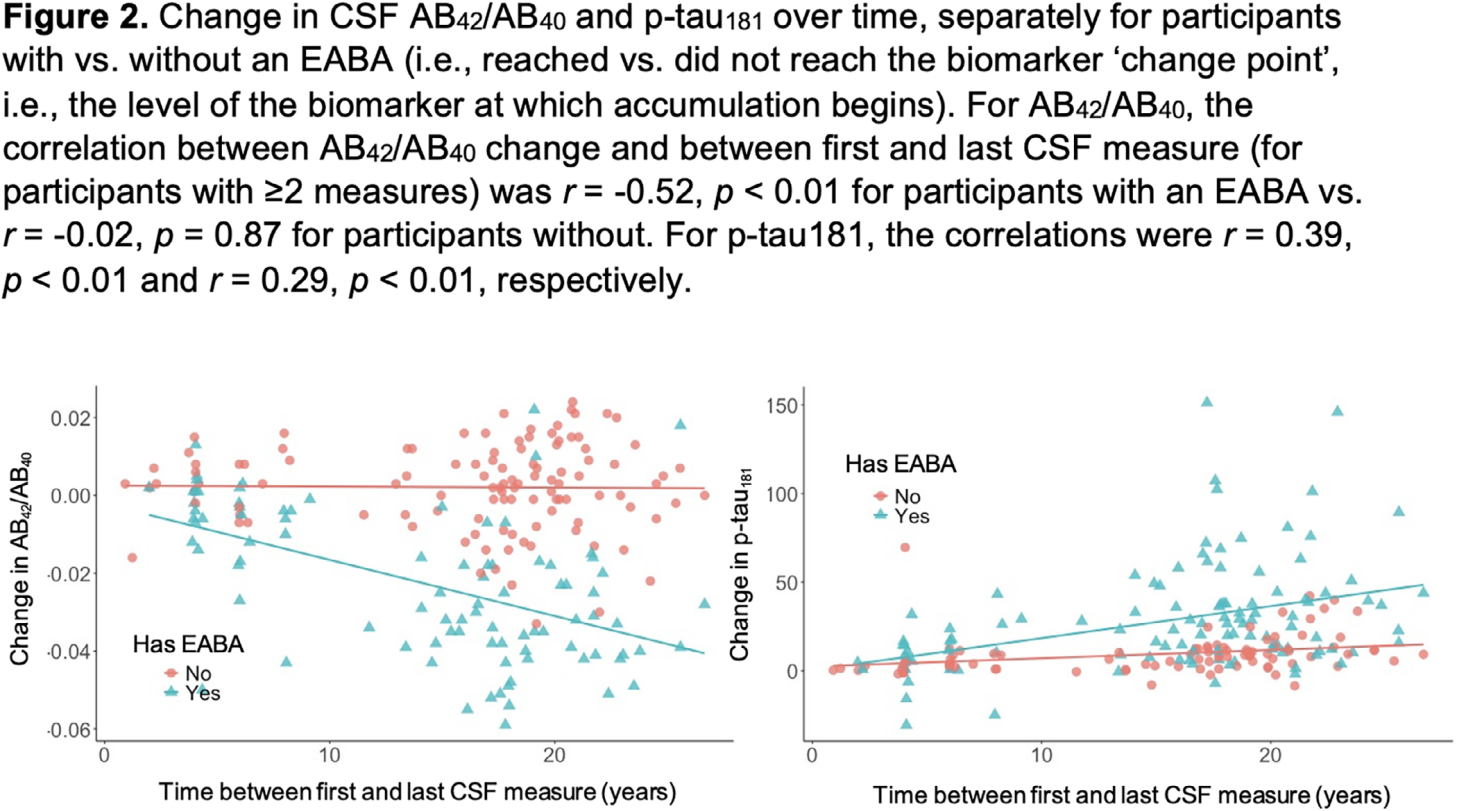# Estimated onset age of CSF amyloid and tau accumulation and relationship to clinical outcomes

**DOI:** 10.1002/alz70856_105770

**Published:** 2026-01-07

**Authors:** Corinne Pettigrew, Yuxin Zhu, Anja Soldan, Mei‐Cheng Wang, Marilyn S. S. Albert, Abhay Moghekar

**Affiliations:** ^1^ Johns Hopkins University School of Medicine, Baltimore, MD, USA; ^2^ Department of Neurology, Johns Hopkins University School of Medicine, Baltimore, MD, USA; ^3^ Johns Hopkins Bloomberg School of Public Health, Baltimore, MD, USA

## Abstract

**Background:**

Neuropathological studies indicate AD pathology is present in midlife. Few studies, however, have examined the onset of AD biomarker changes, simultaneously for amyloid and tau, starting in midlife. Using data from initially cognitively unimpaired, primarily middle‐aged participants, we evaluated: 1) the ‘change point’ that marks accelerated accumulation of CSF AB_42_/AB_40_ and *p*‐tau_181_ and estimated onset ages of biomarker accumulation (EABA); 2) factors impacting EABA variability, and 3) association of EABA with risk of progression to MCI clinical symptoms.

**Method:**

Analyses evaluated CSF AB_42_/AB_40_ and *p*‐tau_181_ (Fujirebio Lumipulse G1200 assays) in *N* = 384 cognitively unimpaired BIOCARD Study participants (*M* baseline age=58.4y, 58% female; *M* = 3.2 CSF measures collected over 8.6y). Biomarker ‘change points’ were identified (in the 62% with ≥2 CSF measures) and EABA calculated for AB_42_/AB_40_ and *p*‐tau_181_ using methods previously applied to amyloid PET (Schindler et al., 2021).

**Result:**

The mean EABA for AB_42_/AB_40_ was 49.3 years vs. 51.6 for *p*‐tau_181_; EABAs were ∼6y earlier for APOE‐e4 carriers vs. non‐carriers (Table 1, Figure 1). Participants with an EABA demonstrated steeper CSF biomarker changes over time, compared to participants without an EABA (Figure 2). In Cox regression models, APOE‐e4 carriers had a shorter time to EABA for both AB_42_/AB_40_ (HR=3.65, *p* <0.01) and *p*‐tau_181_ (HR=2.00, *p* <0.01). In Cox models including the subset of participants with EABAs, older EABAs (but not APOE‐e4, sex, or education) were associated with a shorter time to MCI symptom onset (AB_40_/AB_40_ HR=2.12, *p* <0.01; *p*‐tau_181_ HR=3.34, *p* <0.01); additionally, for AB_40_/AB_40_, the association of EABA with symptom onset accelerated (became increasingly shorter) at older EABAs (HR for EABA^2^=1.35, *p* <0.01). EABA preceded MCI symptom onset by 24 years, on average (SD=8‐11).

**Conclusion:**

Results indicate that AD CSF biomarker changes begin in midlife, decades before MCI clinical symptom onset. They are consistent with the view that both amyloid and tau onset occurs earlier in APOE‐e4 carriers, with additional analyses needed to evaluate whether APOE‐e4‐related changes in *p*‐tau_181_ are due to APOE‐e4‐related AB_42_/AB_40_ changes. Lastly, the shorter time to symptom onset with older EABAs suggest that co‐pathologies (which are greater at older ages) may reduce the time from biomarker accumulation to symptom onset.